# A systematic review of functional near-infrared spectroscopy-based task paradigms in stroke rehabilitation

**DOI:** 10.3389/fnhum.2025.1633142

**Published:** 2026-01-16

**Authors:** Yuping Huang, Xiaoxuan Zhan, Huizi Zeng, Shuyin Li, Jingqin Shi, Zhenhua Cui, Qianqian Fan, Binbin Li, Yanfang Sui, Fengyan Liang, Zhenhua Song

**Affiliations:** 1 School of Rehabilitation Medicine, Shandong University of Traditional Chinese Medicine, Jinan, China; 2 Department of Rehabilitation Medicine, Haikou Hospital Affiliated to Xiangya Medical College of Central South University, Haikou, China; 3 School of Biomedical Engineering, Hainan University, Sanya, China

**Keywords:** brain region, fNIRS, paradigm, rehabilitation, stroke

## Abstract

Precision in assessing neurological function after stroke is key to optimizing the efficacy of rehabilitation. Functional near-infrared spectroscopy (fNIRS) provides a highly ecologically valid assessment of cortical activation and functional reorganization after stroke by monitoring cortical hemodynamic changes during different tasks. However, the current fNIRS task paradigm lacks systematic integration for standardized design and clinical translation strategies, and fragmented evidence is difficult to converge into actionable practice guidelines. To fill this gap, this paper systematically reviews the application of fNIRS in motor, cognitive, language, and dual-task paradigms in stroke rehabilitation research. It reveals the clinical value of different paradigms for neurological function assessment and proposes adaptive task designs that fit the functional characteristics of patients with stroke. This study emphasizes the importance of personalized and ecological paradigms, providing a theoretical basis and practical reference for subsequent standardized research on fNIRS task paradigms and developing clinical application standards.

## 1 Introduction

Stroke, the second most common fatal disease worldwide, accounts for approximately 11.6 million new cases and 5.5 million deaths annually, with more than 50% of survivors left with long-term functional impairment (GBD 2019 Stroke, 2021). The core goal of stroke rehabilitation is to promote functional recovery through neuroplastic remodeling (Singh, 2024), and accurate assessment of functional reorganization of the brain is key to optimizing rehabilitation strategies and achieving individualized interventions. Traditional clinical scales are highly subjective and insufficiently sensitive, creating challenges in capturing early neurological changes; functional magnetic resonance imaging (fMRI), although with high spatial resolution, is environmentally restricted and not applicable to natural rehabilitation monitoring (Chen et al., 2020). In contrast, functional near-infrared spectroscopy (fNIRS) with its portability, resistance to motion artifacts, and high temporal resolution is ideal for stroke rehabilitation assessment (Wang et al., 2025b).

Several stroke rehabilitation studies have used fNIRS. In the field of limb function rehabilitation, fNIRS is shown to detect oxygenated hemoglobin (HbO) concentration in motor networks represented by the sensorimotor cortex (SMC), supplementary motor area (SMA), premotor cortex (PMC), and prefrontal cortex (PFC) during tasks, such as motor imagery (Wang et al., 2022), robotic-assisted training (Li H. et al., 2023; Liu P. et al., 2024), standing balance (Xia Y. et al., 2022), and walking (Lim et al., 2022b). Lee S. H. et al. (2020) observed a balanced cortical activation pattern (decreased activation in SMC and SMA) in patients during the later stages of robot-assisted walking training. In cognitive rehabilitation, functional connectivity decline is considered a promising evaluative indicator for recognizing cognitive dysfunction after stroke (Ai et al., 2025; Zou et al., 2023). Moreover, fNIRS shows that patients with stroke cognitive impairment depend on compensatory activation in the right prefrontal lobe during working memory tasks (Ai et al., 2025). In aphasia, an fNIRS study showed that different interventions could have different pathways to recovery. Head acupuncture combined with speech training significantly increased HbO levels in the left frontal pole region to improve some naming functions (Lin et al., 2025); low-frequency repetitive transcranial magnetic stimulation (rTMS) improved naming performance by decreasing activation in multiple functional areas of language, such as the left superior temporal gyrus (STG), Broca's area, and others (Gan et al., 2024). Together, these studies suggest that fNIRS is uniquely valuable in revealing post-stroke neuroplasticity and optimizing rehabilitation strategies.

Paradigms are crucial to neurofunctional imaging studies (such as fNIRS) to induce the activation of specific brain regions. They include core elements, such as task types, presentation methods, and time parameters (Cao et al., 2021). The scientific design of the task paradigm directly determines the detection ability of neuroimaging tools and their data quality (Zhang et al., 2022), acting as a key link connecting brain signal measurements with the interpretation of clinical significance. However, the significant heterogeneity of stroke patients in terms of motor ability, cognitive function, attention maintenance, and fatigue tolerance poses challenges to designing task paradigms. Standardized tasks may not be adaptable to patients with different levels of dysfunction, resulting in missing data or reduced signal quality; oversimplified tasks may lack sensitivity to detect subtle neurological changes (Zhao et al., 2023). Therefore, designing fNIRS task paradigms that can accommodate patients' functional limitations while maintaining scientific validity is the key issue in neurological function assessment in stroke. Currently, applying the fNIRS task paradigm in stroke rehabilitation faces three major challenges: (1) Designing ecologically valid tasks applicable to patients with different dysfunctions (e.g., alternative exercise programs for hemiplegics); (2) Optimizing the task to support multimodal data integration so that the fNIRS signal can be complementarily validated using electromyography, movement parameters, and other metrics to enhance precise assessment of neural-behavioral associations; (3) Establishing a standardized paradigm library to enhance cross-study comparability and data sharing.

This systematic review will systematically address the research progress of the fNIRS task paradigm in stroke rehabilitation—excluding resting-state measurements—from four aspects: objectives, design principles, classification, and future trends. It will analyze paradigm design strategies based on neuroplasticity mechanisms, adaptive task tuning methods for patients' functional characteristics, and balanced solutions for clinical standardization and individualization. By integrating current evidence and proposing innovative perspectives, it aims to provide systematic guidance to researchers and promote the scientific application of the fNIRS task paradigm in stroke rehabilitation.

## 2 Research methods

**Figure 1:**
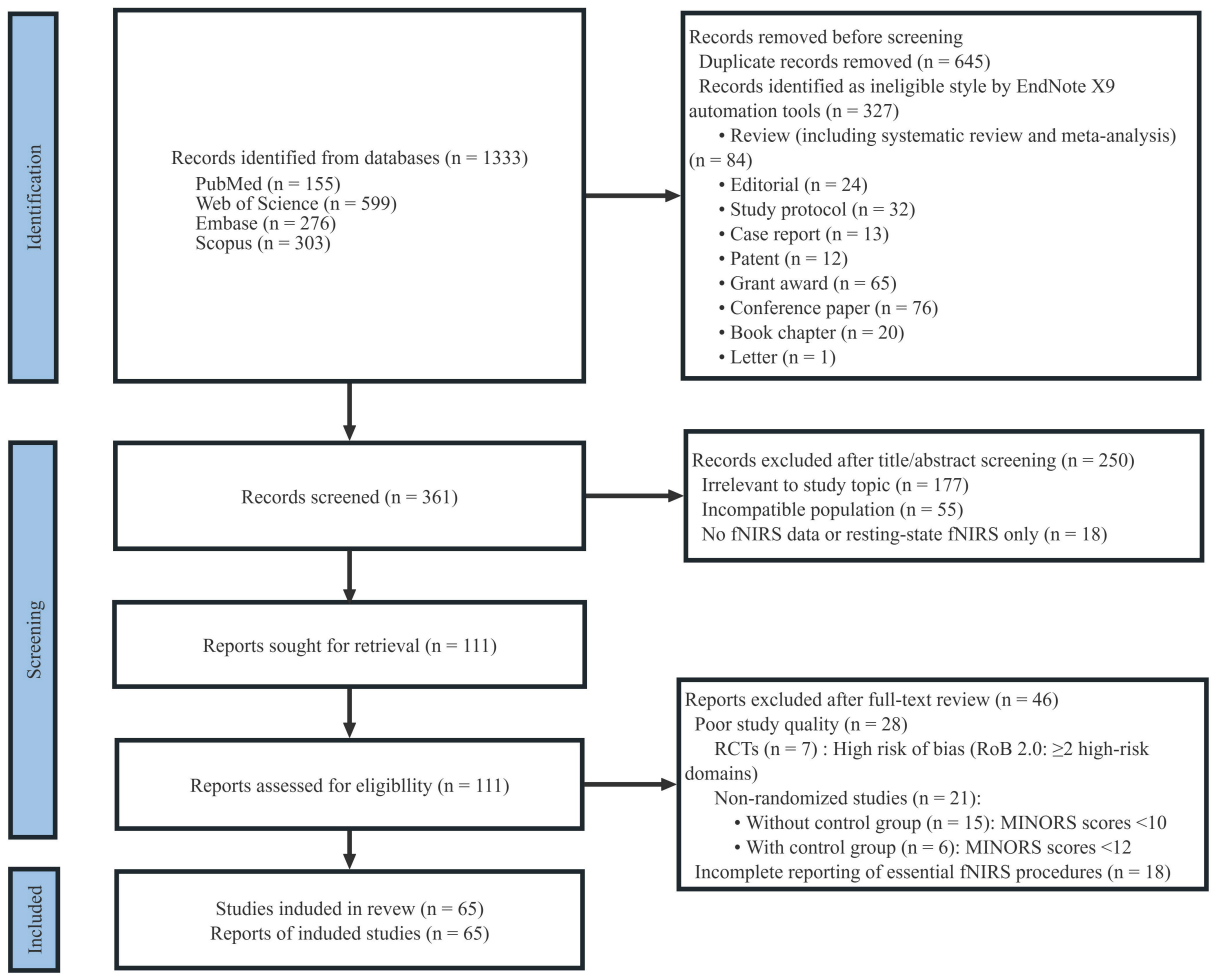
Flowchart of literature search and screening.

The search was completed on February 19, 2025, using PubMed, Web of Science, Embase, and Scopus databases. The subject terms and free terms were mainly obtained from the MeSH database and Emtree and referred to relevant meta-analyses. The search terms “stroke” and “functional near-infrared spectroscopy” ranged from 2019 to 2024 were identified, and the search formula was constructed according to the rules of each database. The literature screening process (Figure 1) followed the Preferred Reporting Items for Systematic Reviews and Meta-Analyses (PRISMA) and was based on the following criteria:

Inclusion criteria:

(1) Stroke patients;
(2) The use of the fNIRS task-state experimental paradigm;
(3) Access to full text and extraction of key information;
(4) Literature in English;
(5) At least one of the following comparisons must be included: stroke patients vs. healthy controls; affected vs. unaffected cerebral regions; pre- vs. post-intervention; or response contrasts across distinct task difficulty levels or task content;
(6) Experimental trials, including randomized controlled trials (RCTs) and non-randomized controlled trials (non-RCTs), and observational studies.

Exclusion criteria:

(1) Not in style—review (including systematic review and meta-analysis), editorial, study protocol, case report, patent, grant award, conference paper, book chapter, letter;
(2) Irrelevant to the study topic (e.g., non-stroke subjects, non-rehabilitation studies, not using fNIRS or collecting resting-state data only);
(3) Poorly assessed study quality;
(4) Unavailability of full text or missing key information.

For included studies, key information was extracted: this included the content of the experimental paradigm based on the fNIRS task, fNIRS metrics (e.g., ΔHbO), regions of interest (ROIs), metrics for statistical analyses, and tools or procedures used for data processing and analysis.

## 3 Results and discussion

This review included 65 papers (42 non-randomized studies and 23 randomized controlled trials). The fNIRS paradigms used in the research were based on motor (67.69%), cognitive (12.31%), language (4.62%), swallowing (7.69%), and dual-task (7.69%). Table 1 lists the task design and brain regions of interest in the included studies. All studies were evaluated for quality using the Cochrane Risk of Bias 2.0 tool (RoB 2.0) for RCTs (Page et al., 2021), and the Methodological Index for Non-Randomized Studies (MINORS) (Slim et al., 2003) for non-randomized studies. Despite the exclusion of high-risk studies during the screening process, the majority of RCTs still demonstrated a high risk of bias. The primary sources of bias were identified in the areas of blinding, data integrity, and allocation concealment. Among the 13 non-randomized studies without a control group, all were classified as moderate risk, with MINORS scores ranging from 10 to 13. The main sources of bias in these studies included insufficient blinding, high attrition rates, and a lack of sample size calculation. In the 29 non-randomized studies with control groups, 75.86% were categorized as moderate risk (total score between 15 and 19), 13.79% as low risk (total score ≥20), and 10.34% as high risk (total score < 15). The predominant sources of bias in these studies were high attrition rates, inadequate sample sizes, and baseline inequivalence between groups. These issues significantly affected the reliability and generalizability of the results. Overall, while the included studies met basic quality standards, improvements are needed in areas such as blinding, attrition management, and sample size calculation to enhance the quality and reliability of future research.

**Table 1:** Task design and brain regions for included studies.

**Included studies**	**Task design**	**Brain regions**	**Included studies**	**Task design**	**Brain regions**	**Included studies**	**Task design**	**Brain regions**
Wang et al., 2022	Dual-task	SMC	Muller et al., 2024	Motor	SM1	Chang P. W. et al., 2022	Motor	M1, SMA
Collett et al., 2021	Dual-task	PFC	Dai et al., 2024	Motor	PFC, PMC&SMA	Bello et al., 2021	Motor	M1, PCu
Sakurada et al., 2019	Dual-task	PFC	Wang M. H. et al., 2024	Motor	PFC, SMC	Jian et al., 2021b	Motor	PFC, M1
Chatterjee et al., 2019	Dual-task	PFC	Ye et al., 2024	Motor	PMC, M1, S1	Jian et al., 2021a	Motor	PFC, M1
Nosaka et al., 2023	Dual-task	FPC	Cheng et al., 2024	Motor	PFC, SFC, SMA, PMC	Bello et al., 2020	Motor	M1, PCu
Yu H. et al., 2024	Cognitive	DLPFC, FPC, STG, OFC	Liu P. et al., 2024	Motor	PFC, MC, OC	Bai and Fong, 2020	Motor	DLPFC, PMC, SMA, M1, S1
Liu Y. et al., 2024	Cognitive	PFC (DLPFC, FPC, OFC, IFGtri)	Chen Y. F. et al., 2023	Motor	PMC, SMA, SM1, SAC	Kinoshita et al., 2019	Motor	M1, PMC&SMA
Kong et al., 2023	Cognitive	PFC, SMC	Bu et al., 2023	Motor	PFC, MC, OC	Lim and Eng, 2019	Motor	SMC
Liu Y. et al., 2023	Cognitive	DLPFC, PMC, SM1	Huo et al., 2023	Motor	PFC, MC	Delorme et al., 2019	Motor	SM1
Tetsuka et al., 2023	Cognitive	DLPFC, FPC	Li H. et al., 2023	Motor	PFC, PMC&SMA, M1, S1	Ma et al., 2025	Motor	PFC, PMC&SMA, M1, S1, SMG
Li X. et al., 2023	Cognitive	PFC	Chu et al., 2023	Motor	PFC, PMC, SMA, M1, SMC	Tamashiro et al., 2019	Motor	SM1, PMC&SMA
Chu et al., 2022	Cognitive	DLPFC, FPC, Broca area	Ma et al., 2023	Motor	PFC, SMC, OC	Zou et al., 2024	Motor	PFC, MC, OC
Yang et al., 2022	Cognitive	mPFC, DLPFC, VLPFC, SFC, STC	Kim et al., 2023	Motor	M1, PMC, SMA	Liu L. et al., 2022	Motor	MC, FC
Gan et al., 2024	Language	DLPFC, STG, MTG, SMA, Broca area, Wernicke area	Chen S. et al., 2023	Motor	M1, PMC, SMA	Chen et al., 2024	Motor	SMC
Xie et al., 2022a	Language	PFC, MC, OC	Wang L. et al., 2023	Motor	DLPFC, FPC, OFC, S1, PMC&SMA, M1, Wernicke area, Broca area	Yang et al., 2024	Motor	DLPFC, M1, SMA
Gilmore et al., 2021	Language	SFC, MFG, IFGtri, IFGoper, PCG, MTG, SMG, AG	Shin et al., 2022	Motor	SM1, PMC, SMA	He et al., 2023	Motor	PMC, SMA, M1, S1
Ma X. et al., 2024	Swallowing	PFC, S1, M1, PMC&SMA	Guo et al., 2022	Motor	PFC, M1, S1	Heiberg et al., 2023	Motor	PFC
Wen et al., 2023	Swallowing	PFC, PMC&SMA, S1	Ma et al., 2022	Motor	PFC, MC, OC	Lim et al., 2022b	Motor	PFC, PMC, SMC, PPC
Fu et al., 2023	Swallowing	PFC, PMC&SMA, M1, S1	Xia W. et al., 2022	Motor	PMC, SMA, SMC	Caliandro et al., 2020	Motor	PFC
Liu H. et al., 2022	Swallowing	PFC, MC, OC	Yuan et al., 2022	Motor	PMC, SMA, SM1	Lee A. et al., 2020	Motor	SMC
Wang et al., 2024	Swallowing	PFC, PMC&SMA, M1, S1, rSMG	Xu G. et al., 2022	Motor	PFC, MC	Xia Y. et al., 2022	Motor	M1, PMC, SMA
Kim et al., 2024	Motor	PFC, SMA, M1, S1, PPC	Kim H. et al., 2022	Motor	M1, PMC, SMA, S1			

Abbreviations for the brain regions: AG, angular gyrus; DLPFC, dorsolateral prefrontal cortex; FC, frontal cortex; FPC, frontopolar cortex; IFGoper, inferior frontal gyrus pars opercularis; IFGtri, inferior frontal gyrus triangular part; M1, primary motor cortex; MC, motor cortex; MFG, middle frontal gyrus; mPFC, medial prefrontal cortex; MTG, middle temporal gyrus; OC, occipital cortex; OFC, orbitofrontal cortex; PCG, precentral gyrus; PCu, precuneus; PFC, prefrontal cortex; PMC, pre-motor cortex; PMC&SMA, premotor cortex and supplementary motor area; PPC, posterior parietal cortex; S1, primary somatosensory cortex; SAC, somatosensory association cortex; SFC, superior frontal cortex; SM1, primary sensorimotor cortex; SMA, supplementary motor area; SMC, sensorimotor cortex; SMG, supramarginal gyrus; STC, superior temporal cortex; STG, superior temporal gyrus; VLPFC, ventrolateral prefrontal cortex.

### 3.1 The objective of the fNIRS task paradigm

The fNIRS task paradigm aims to activate key brain regions through specific task design to dynamically resolve post-stroke neuroplasticity and optimize the objective basis of neurological level for rehabilitation strategies. Compared with traditional resting-state functional imaging, fNIRS can realize the transition from static observation to dynamic functional analysis by inducing activation of brain regions through task settings and capturing functional reorganization and compensation in real time. For example, the motor imagery (MI) task mainly activates the PFC and SMC (Kotegawa et al., 2020; Yu Y. et al., 2024; Hurst and Boe, 2022), the motor execution task significantly activates the primary motor cortex (M1) (Jalalvandi et al., 2024), and the cognitive task highlights the involvement of the PFC and parietal lobe (Heiberg et al., 2023). Different task paradigms can map neural network surrogate pathways: simple repetitive tasks (e.g., ankle dorsiflexion) are prone to standardization but underestimate network reorganization (Liang et al., 2022); complex dual-tasks (e.g., walking combined with mental arithmetic) expose deficits in the allocation of resources to the PFC (Wang Q. et al., 2023); ecological tasks such as virtual reality (VR) tasks activate a wider range of cortical networks (Parker et al., 2024). By rationally designing tasks, fNIRS parses neuroplasticity multidimensionally and provides a basis for assessing intervention effects and guiding personalized rehabilitation, making it a valuable tool for dynamic neurofunctional mapping.

### 3.2 Basic principles of task paradigm design

#### 3.2.1 Time parameter design

The fNIRS task paradigm is mainly divided into two categories: block design and event-related design. The block design is the preferred paradigm for stroke research due to its high signal-to-noise ratio, and it obtains stable HbO and deoxygenated hemoglobin (HbR) signals by focusing on the presentation of similar stimuli for 20–30 s (Luke et al., 2021). In contrast, event-related designs use short 2–5 s stimuli to capture transient responses (Defenderfer et al., 2017). Based on hemodynamic response characteristics, a standard cycle of a 20–30-s task period (up to the oxygenation plateau) with a 30–40-s baseline period is recommended (Herold et al., 2017, 2018). In older stroke patients or those with poor functional status, appropriately prolonged rest periods (at least 45 s) can effectively minimize fatigue effects and cumulative disturbances in the blood oxygenation response (Herold et al., 2018). When assessing brain network function, acquisitions longer than 4 min ensure stability and reliability of core metrics (Xu et al., 2023). The motor task requires 5–8 repetitions to obtain a stable signal, which may result in an excessively long total experimental time. The total experiment time is usually 15–20 min, with additional resting sessions when necessary to balance signal quality and patient attention duration. Most studies have adopted a “20-s task-30-s rest” pattern, repeated five times (Shen et al., 2024), to balance the total task time and data reliability. The design of experimental paradigms should account for individual differences in patients, including cognitive function, age, and pathological characteristics, to improve the scientific validity of fNIRS studies through individualized paradigm adjustments.

In event-related design, each trial is treated as a discrete event. The inter-stimulus interval (ISI) is typically set between 2 and 6 s (Geissler et al., 2021). It is recommended to use jittered interstimulus intervals or longer ISIs to ensure that the hemodynamic response function (HRF) for each event is not contaminated by adjacent stimuli. Randomized ISIs help reduce the effects of predictability and fatigue, thereby increasing statistical power (Jeong et al., 2025). The duration of the stimulus needs to match the characteristics of the stimulus conditions and the objectives of the experiment. For example, the Stroop task has a stimulus duration of 500 milliseconds (Li B. et al., 2024) to 2 s (Schroeter et al., 2002), while a more complex semantic task requires 5 s (Gilmore et al., 2021). This is because the former assesses quick reaction capabilities, while the latter requires participants to engage in semantic processing and information integration. The repetition frequency within a single trial is typically conducted 20 times (Geissler et al., 2021; Li B. et al., 2024) for each condition to ensure reliable signal averaging. The number of trials is determined by the specific research requirements and the sensitivity of the fNIRS system. Compared to block design, the event-related design of fNIRS paradigms is more suitable for exploring the instantaneous responses of brain regions to specific tasks. Therefore, event-related design is more appropriate for the developmental research of intervention technologies such as brain-computer interfaces and neurofeedback, which require real-time capabilities.

#### 3.2.2 Stimulus presentation method

Stimulus modality selection should incorporate the perceptual characteristics of patients with stroke. Visual stimuli and auditory instructions are most commonly used in fNIRS studies. Auditory instructions should be short and clear to avoid increasing cognitive load (Potts et al., 2024; Peng et al., 2023). In practice, researchers tend to use multimodal stimulation (combining audio and visual) to improve task comprehension. Instruction design is a key aspect of fNIRS research in stroke. Patients with stroke often exhibit slowed information processing (Lugtmeijer et al., 2021); therefore, instruction design should follow the principle of simplification and use single-step instructions in the acute phase instead of multi-step instructions. Using the “demonstrate-practice-execute” model before starting a trial helps patients understand task requirements and significantly reduces the rate of data abandonment (Seitz et al., 2024). Feedback mechanisms are crucial to maintaining patient engagement (Shin and Chung, 2022; Kim et al., 2025), with immediate feedback facilitating correct task performance and delayed feedback favoring long-term learning (Palidis et al., 2025). Combining multiple feedback modalities can effectively stimulate the sensory system and promote the reconstruction of motor function and neuroplasticity (Rendos et al., 2021; Yuan et al., 2021; Noh et al., 2019; Matarasso et al., 2021). Combining neurofeedback tasks improves SMC activation in the affected hemisphere (Mihara et al., 2021), which is particularly important for rehabilitation in the chronic phase. Stimulus intensity can be designed in three ways: fixed difficulty, progressive enhancement, or adaptive design (Maier et al., 2019). Fixed difficulty is suitable for standardized assessment, progressive designs (e.g., from single to multiple joint movements) are suitable for training, and adaptive designs (adjusting difficulty based on real-time performance) are best for individualized rehabilitation (Ma et al., 2023; Matarasso et al., 2021; Rieke et al., 2020). Difficulty designs that generally aim for a 70–80% success rate strike a balance between challenge and frustration to optimize rehabilitation. fNIRS assessments can dynamically adjust task difficulty for blood oxygenation signals to avoid over-activation or under-response (Huo et al., 2022; Kohl et al., 2020).

#### 3.2.3 Adaptive task design for stroke victims

Stroke, as a complex neurological disease, poses unique challenges for fNIRS task paradigm design. Patient motor dysfunction, cognitive impairment, and susceptibility to fatigue affect assessment feasibility, reduce data quality degradation, and trigger bias (Skau et al., 2021; Pan et al., 2019; Csípo et al., 2021; Takahashi et al., 2021). To address these challenges, adaptive task design can better accommodate patient functional differences while maintaining neuroscience rigor. The core concepts include: first, “feasibility first” to ensure that most patients can complete the assessment and avoid sample bias due to screening for functional level; second, “information maximization” to obtain as much valid neurological data as possible, even if the task is simplified. Achieving these two goals requires researchers to make strategic trade-offs in trial design. Three main adaptation strategies have been developed to address motor function limitations (Guo et al., 2022; Qu et al., 2025; Almulla et al., 2022; Su et al., 2023; Kotegawa and Teramoto, 2022): the healthy-side substitution task: by observing the effect of healthy-side movement on the affected neural network, the complex relationship between neural inhibition and facilitation across hemispheres is explored in depth, which helps to understand the potential mechanisms of neural reorganization after brain injury; the passive-motor paradigm: the activation of sensorimotor networks using an external assistive device, which provides an opportunity for severely limited motor ability; motor imagery tasks: indirectly assessing motor function and neuroplasticity through internal neural activation of motor preparation and planning networks. These three strategies cannot replace traditional tasks, but can reflect the key features of motor network reorganization from different perspectives.

Adaptive design for cognitive impairment is based on reducing non-target cognitive load (Bijarsari, 2021). By dynamically adjusting task difficulty so that the task is always in the patient's “comfort zone,” the frustration caused by a task that is too difficult and the ceiling effect caused by a task that is too easy are avoided. Extended reaction time and multimodal cues compensated for the decline in cognitive processing efficiency in both the temporal and perceptual dimensions, ensuring that the task was focused on cognitive functioning instead of processing speed or attention maintenance (Bello et al., 2021).

Regarding fatigue management, the adaptive design shifts from a “single continuous measure” to a “chunked cumulative assessment.” Short time chunking reduces the physical burden on the patient and improves signal quality by reducing interference from head movements and physiologic drift. Real-time load monitoring introduces the concept of adaptive measurement and dynamically adjusts task load based on neural indicators, which is superior to subjective reports (Matarasso et al., 2021; Kohl et al., 2020; Asgher et al., 2021). Gamification tasks combat fatigue by stimulating interest, enhancing ecological validity (Yu et al., 2022; Bae and Park, 2023). Individualized calibration and ability matching further safeguard the scientific and ethical nature of the study. Adaptive task design should focus on adaptive algorithm development, standard task library construction, and multimodal integration in the future to promote accurate personalized assessment.

### 3.3 Classification of task paradigms in stroke rehabilitation

#### 3.3.1 Paradigms related to motor function rehabilitation

##### 3.3.1.1 Active movement tasks

Active motor tasks, the most widely used paradigm in fNIRS stroke research (Table 2), are commonly used to explore the neural mechanisms of functional recovery of the upper limb (Xu G. et al., 2022; Lu et al., 2023; Ni et al., 2023; Xu R. et al., 2022; Du et al., 2022; Bonnal et al., 2024; Li C. et al., 2022). Most studies have assessed the activation status of the motor cortex on the affected side using a block design with specific motor paradigms (Lim and Eng, 2019; Yang et al., 2024; He et al., 2023; Mihara et al., 2021; Bonnal et al., 2023; Borrell et al., 2023; Zhao et al., 2024; Lim et al., 2021): the upper limb paradigm (grasping, finger-pairing, and picking up) may reflect fine-motor abilities with sensorimotor integration; the lower limb paradigm (standing, walking, and ankle dorsiflexion) focuses on balance and coordination, with the walking task having high ecological validity. Overall, these designs are simple and easy to standardize, but their relevance to everyday functioning remains to be strengthened.

**Table 2:** Classification of active movement task designs.

**Type of movement task**	**Difficulty level**	**Typical actions**	**Applicable patient stages**	**Characteristics of neural activation**
Single-joint repetition (Liang et al., 2022; Chen N. et al., 2023)	Low	Finger tapping, ankle dorsiflexion	Acute phase	Local motor area activation
Multi-joint coordination (Chen N. et al., 2023; Lacerenza et al., 2023; Li C. et al., 2024)	Medium	Grasping, walking	Recovery phase	Activation of motor and sensory networks
Functional combination (Matsuo et al., 2021)	High	Simulating daily-life activities	Sequelae phase	Activation of a wide range of cortical networks

With a deeper understanding of neural plasticity, active motor task designs have evolved from single movements to task sequences, expanding from unilateral assessments to bilateral comparisons, and gradually introducing feedback interactions (Muller et al., 2024; Xu G. et al., 2022; Parker et al., 2024; Bae and Park, 2023; Du et al., 2022). VR/augmented reality (AR) technology enhances executive motivation through immediate visual feedback and, more importantly, creates closed-loop neurofeedback that integrates sensory, cognitive, and motor network assessment, breaking the limitations of traditional paradigms (Liu P. et al., 2024; Cui et al., 2025; Taguchi et al., 2022).

The design of active motor tasks should account for the patient's functional status and stage of recovery (Ma et al., 2023). Simple, low-intensity movements are preferred in the acute phase, and complexity can be gradually increased in the recovery phase; goal-oriented tasks enhance cortical activation (Lacerenza et al., 2023). This hierarchical design enhances data reliability and allows for continuous monitoring of the entire rehabilitation process. Moreover, safety is a primary consideration for patients with stroke performing motor tasks (especially walking paradigms), and suspension systems reduce the risk of falls (He et al., 2023). The active motor task paradigm should focus on individual differences, extend laboratory-standardized movements to functional tasks of daily living, and transition from single-assessment to longitudinal dynamic monitoring to enhance the clinical applicability of the fNIRS assessment and provide a strong neuroscientific support for precision rehabilitation.

##### 3.3.1.2 Passive movement tasks

A passive motor task guided limb movements in patients with severe stroke by external forces and recorded cortical activation of sensory inputs, demonstrating that sensory pathways drive motor networks even in a paralyzed state (Cheng et al., 2024). This finding provides an important theoretical basis for early rehabilitation from a neuroscientific perspective-the integrity of sensory pathways may be a prerequisite for motor function recovery. Unlike active movements that primarily activate M1 and SMA, passive movements focus on the sensory cortex and premotor area (PMA) (Li et al., 2024). Robot-assisted technology has improved the standardization (Liu P. et al., 2024; Dai et al., 2024; Jiang et al., 2022) and data reliability (Xie et al., 2022b; Bonanno et al., 2023) of passive tasks, facilitating longitudinal monitoring of the rehabilitation process.

In clinical practice, passive motor tasks have a “dual role.” They assess the potential for sensory-motor integration and recovery and activate residual neural networks to prevent disuse atrophy through sustained passive activity. Future studies should focus on the neural mechanisms of passive adaptation to active remodeling to provide precise neurobiological markers for clinical rehabilitation grading. Passive motor tasks should be used as an alternative to active movement and guide patients from passive perception to active movement through sensory feedback to achieve true functional rehabilitation.

##### 3.3.1.3 Motor imagery task

The unique value of the motor imagery task is that it builds a neural bridge of “intention-action” for patients who cannot perform actual movement but have preserved cognitive function (Hurst and Boe, 2022; Villa-Berges et al., 2023; Wang H. et al., 2023; Moran and O'Shea, 2020; Mehler et al., 2020). Unlike active or passive movements, MI does not depend on executive ability to activate the motor preparation and planning loop, which induces SMA/PMC and, in some patients, activation of the M1, the level of which strongly correlates with recovery potential (Mihara et al., 2021; Wang et al., 2025a).

Improvement in MI task effectiveness relies on fine-tuning the design. Studies have shown that externally-supported imagery (e.g., VR) is more effective than imagery alone (Choy et al., 2023; Kim D. H. et al., 2022); multisensory guidance (e.g., movement observation, sound rhythms) produces a stronger cortical response than a single instruction (Almulla et al., 2022; Errante et al., 2022; Eaves et al., 2024; Choi et al., 2022); graded-difficulty designs can be adapted to the imagery abilities of different patients (Ji et al., 2021; Wriessnegger et al., 2017); dominant hand tasks are more likely to induce strong activation, non-dominant hand imagery should be more vivid (Wang et al., 2025a). Regarding clinical translation, integrating MI tasks with brain-computer interface (BCI) systems is a new avenue for stroke rehabilitation (Batula et al., 2017; Wang et al., 2023; Ma Z. Z. et al., 2024; Khan et al., 2020). The fNIRS-based MI-BCI system helps patients adjust their imagery strategies through real-time feedback, reinforcing the activation of specific brain regions and activating more neural circuits than conventional training, especially the key pathways connecting intention and action (Lin et al., 2022; Liu X. et al., 2023).

Despite their advantages, MI tasks face challenges in objectively monitoring the quality of execution. Whether or not the patient actually performs the MI and the quality of imagery both directly affect the reliability of results. Studies have shown that false-positive feedback significantly reduces cortical activation during training in subjects, especially in contralateral motor areas, which can negatively impact cortical plasticity (Jeong et al., 2025). Therefore, a more accurate physiological index assessment system should be constructed using electromyography and other monitoring tools (Qin et al., 2025) to achieve quality control and enhance cortical activation and neuroplasticity. The MI task should be optimized through personalized customization, multimodal fusion, and closed-loop feedback to improve network activation and be linked with external assistive devices to build an “intention-execution-feedback” closed-loop, which provides dynamic support for stroke rehabilitation.

#### 3.3.2 Cognitive tasks

Cognitive dysfunction is a common and prognostic problem after stroke, involving several core components such as working memory, inhibitory control, cognitive flexibility, and planning ability (Schumacher et al., 2019). fNIRS commonly uses three types of classical cognitive paradigms (Lu et al., 2025; Cheng X. P. et al., 2024; Udina et al., 2020): the verbal fluency task test (VFT) is used to assess verbal initiation and executive search ability; the n-back task targeting working memory capacity and updating function; the Stroop task assessing cognitive inhibitory control. Reduced PFC activation, interhemispheric balance dysregulation, and decreased efficiency of network integration are three neurophysiological features of executive dysfunction after stroke (Zou et al., 2023; Udina et al., 2020; Li X. et al., 2022). Results of cross-sectional studies have shown that the magnitude of PFC blood oxygenation signaling changes in cognitive tasks is significantly lower in patients with stroke than in healthy controls, and the attenuation is more pronounced with increased task difficulty (Kong et al., 2023; Csípo et al., 2021; Sunwoo et al., 2023; Sun et al., 2022; Huang et al., 2024). This underactivated performance was significantly correlated with clinical executive function scores (Ai et al., 2025; Li X. et al., 2023), supporting the feasibility of fNIRS as a marker of neurological function. Longitudinal studies have further found that patients in the early stages of stroke tend to show a compensatory response of PFC overactivation; as recovery progresses, the activation pattern gradually normalizes, and improvements in neurophysiological markers usually precede the recovery of behavioral function (Zou et al., 2023; Kong et al., 2023). This trajectory of change suggests a critical time window for rehabilitation interventions. To improve the ecological validity of measures and sensitivity to mild cognitive impairment, assessment paradigms are shifting from traditional single-tasks to more realistic designs such as multitask switching, complex problem solving, and simulation of daily activities in virtual reality environments to identify cognitive deficits at an earlier stage and guide rehabilitation interventions.

#### 3.3.3 Language tasks

Language processing consists of four levels: phonological, syntactic, semantic, and pragmatic. The complexity of language dysfunction arises precisely from the multicomponent nature of language processing (Varkanitsa and Kiran, 2022; Stefaniak et al., 2021). fNIRS has a unique advantage in assessing post-stroke language function, as it allows patients to complete language tasks in a natural communicative environment, reducing the interference of motion and noise with language measurements that occurs with traditional neuroimaging techniques. Early studies validated the reliability of monitoring activation in classical language areas (Broca's and Wernicke's areas) through naming and word generation tasks (Gan et al., 2024; Hara et al., 2017). Subsequently, studies have progressively refined the assessment of the components of language functioning, developing specialized paradigms for different segments: naming tasks reflect the neural basis of patients' lexical extraction, semantic processing, and linguistic expression (Gilmore et al., 2021; Chang W. K. et al., 2022); semantic and phonological fluency tasks assess lexical extraction ability (Gilmore et al., 2021; Guo et al., 2025; Zhang et al., 2023; Fujii et al., 2021); bilingual switching tasks probe language control mechanisms (Farrukh et al., 2025). Recent studies have further revealed the critical role of the dorsal motor cortex in implicit speech, expanding the understanding of the functional connectivity of brain regions involved in language control (Si et al., 2021). In the clinical setting, patterns of PFC activation during acute-phase language tasks predict long-term recovery. Neuroindicators combined with traditional language assessment help create more accurate prognostic prediction models for early rehabilitation planning (Butler et al., 2020).

Current fNIRS language function assessment tasks still have significant limitations. Most paradigms are based on English, posing challenges in generalizing them to language systems, such as Chinese, that differ significantly in morphology, phonology, and semantic structure. Moreover, these tasks focus on the lexical and sentence levels and lack a holistic assessment at the dialog and chapter levels. The analysis focuses on activating local brain regions, neglecting the synergistic effect of the whole brain functional network during language processing. Future assessments of language function need improvements in several directions. For example, developing natural language tasks with higher ecological validity to cover multi-level processing contexts from words and sentences to real conversations and narratives; designing tasks specific to different language characteristics to better reflect the structure and function of each type of language; combining functional connectivity analyses to expand the scope of assessment from local brain area activation to the dynamics of the whole brain network. These improvements will enable the fNIRS language function assessment to integrate basic and clinical research and facilitate the development of personalized language rehabilitation programs based on neural mechanisms.

#### 3.3.4 Swallowing tasks

Swallowing dysfunction is common after stroke and seriously affects the nutritional intake and quality of life in patients (Labeit et al., 2023). fNIRS is more commonly used for limb dysfunction and less for swallowing function (Gallois et al., 2022), probably because swallowing involves multiple regions of the brainstem and cortex and complex sensory-motor coordination, and the brainstem is a key control center. fNIRS is limited by its ability to detect deep brain regions (e.g., brainstem), posing challenges in capturing the activity of these core brain regions. Current fNIRS swallowing task paradigms typically include various designs, such as salivary, autonomous, and commanded swallowing (Ma X. et al., 2024; Wen et al., 2023; Wang et al., 2024; Matsuo et al., 2021) that reveal the functional state of swallowing-related neural networks by evoking specific cortical activation patterns. Recent studies have used a paradigm closer to everyday life, in which subjects are asked to complete sequential movements of gripping, chewing, and swallowing (Matsuo et al., 2021).

Studies have shown that swallowing tasks primarily activate the primary somatosensory cortex, motor cortex, frontal regions, and brainstem association areas (Ma X. et al., 2024; Wen et al., 2023). Swallowing task design should consider safety and feasibility, and rationally set the number of swallows, time interval, and command mode to avoid the risk of aspiration and fatigue. Moreover, similar cortical activation patterns are observed for imagined swallowing in patients who cannot perform the swallowing maneuver (Matsuo et al., 2021).

#### 3.3.5 Dual-task design

The dual-task paradigm provides a measure closer to real-life scenarios for functional assessment of patients with stroke by simultaneously observing the interaction of cognitive and motor tasks (Stephens et al., 2023). Compared with a single task (Table 3), the dual-task paradigm can more sensitively detect subtle changes in early functional deficits and rehabilitation, revealing potential problems that are difficult to detect with single-task assessment (Chiaramonte et al., 2022; Lindberg et al., 2024; Ohzuno and Usuda, 2019). Based on the resource competition theory, cognitive and motor control systems share limited attentional resources, and the efficiency of their resource allocation determines dual-task performance (Strobach, 2020; Tsang et al., 2022; Strobach, 2024). Patients with stroke often show significant functional deficits and increased energy cost of walking (Cw) in dual-task conditions due to reduced processing resources and decreased allocation efficiency as a result of brain damage (Compagnat et al., 2023; Muci et al., 2022; Nonnekes et al., 2020).

**Table 3:** Summary of fNIRS single-task paradigms in stroke rehabilitation.

**Task type**	**Characteristics**	**Advantages**	**Limitations**	**Applicable stages**
Active movement tasks (Muller et al., 2024; Bu et al., 2023; Ma et al., 2025; Chu et al., 2023; Chen et al., 2024)	Block design; mainly focused on upper-limb functions	Directly assess the activation of the motor cortex; observe the balance between hemispheres; easy to standardize	Insufficient relevance to daily life; difficult to cover complex movements; large individual differences	Recovery–chronic phases
Passive movement tasks (Li H. et al., 2023; Liu P. et al., 2024; Zou et al., 2024; Ma et al., 2022)	Guided by external forces; focus on sensory input	Suitable for severely ill patients; verify the integrity of the sensory pathway; minimal movement requirements	Lack of active participation; single activation pattern; limited transfer effect	Acute phase
Motor imagery tasks (Li H. et al., 2023; Cheng et al., 2024)	Simulate intentional movements; activate the prefrontal and sensorimotor cortices	No movement restrictions; can be intervened early; comprehensive activation of neural networks	Rely on patients' imagination abilities; significant individual differences; difficult to quantify	All rehabilitation stages
Cognitive tasks (Yu H. et al., 2024; Liu Y. et al., 2024; Kong et al., 2023; Liu Y. et al., 2023; Tetsuka et al., 2023; Li X. et al., 2023; Chu et al., 2022)	Involve memory, inhibitory control	Evaluate cognitive functions from multiple dimensions; high sensitivity; detect early cognitive changes	Limited task design lack of ecological validity; poor cross-language applicability	Recovery phase
Language tasks (Gan et al., 2024; Xie et al., 2022a; Gilmore et al., 2021)	At the phonetic, grammatical, semantic levels	Reduce environmental interference; evaluate language functions at multiple levels; in a natural communication environment	Mostly designed based on English; lack of dialogue and discourse assessment	Recovery phase
Swallowing tasks (Ma Z. Z. et al., 2024; Wen et al., 2023; Fu et al., 2023; Liu H. et al., 2022; Wang et al., 2024)	Involve the coordination of multiple brain regions	High ecological authenticity; assess sensorimotor integration; simulate daily eating activities	Difficult to detect deep-brain regions; high safety requirements; large individual differences	Early–recovery phase

PFC was over-activated in dual-task conditions in patients with stroke and was significantly associated with execution costs (e.g., longer completion time, decreased accuracy), reflecting neural compensatory mechanisms. While different types of cognitive tasks affected motor performance differently, the interference produced by executive function tasks (e.g., working memory) was more pronounced, revealing the critical role of executive control networks in cognitive-motor integration. Dual-task training reduces PFC activation and improves behavioral performance, supporting the “neuroefficiency” hypothesis that training enhances the efficiency of resource allocation (Nosaka et al., 2023; Wang Q. et al., 2023; Sun et al., 2022; Compagnat et al., 2023; Bishnoi et al., 2021; Ding et al., 2024; Ou et al., 2024; Lim et al., 2022a).

Although the dual-task paradigm provides a more relevant measure for stroke rehabilitation assessment, it still suffers from the lack of standardization, limited comparability of results, unclear effects of heterogeneity in brain injury type on task performance, and neglect of whole-brain network synergies, as most studies concentrate on PFC. To this end, future work should focus on advancing the following directions: establishing a standardized dual-task assessment system with graded difficulty to meet the needs of patients with different levels of functioning; expanding the coverage of brain regions to achieve systematic research on the synergistic mechanism of multiple brain regions, such as motor area and parietal lobe; constructing a prediction model of dual-task performance and daily ability by combining with real-life functional indexes to enhance the value of clinical translation. The dual-task paradigm is a bridge that connects experimental research and clinical application and is expected to promote functional recovery in patients with stroke in complex environments. Future research should strengthen its ecological validity and practicality, helping the dual-task paradigm become an important basis for decision-making in stroke rehabilitation.

### 3.4 fNIRS and clinical metrics

fNIRS quantifies cortical activity by measuring the concentration changes of HbO and HbR, with HbO serving as the primary proxy for neurovascular coupling. Task-related hemodynamic responses are typically expressed as beta coefficients derived from the general linear model (GLM), which convolves the experimental design with an assumed hemodynamic response function (Yu H. et al., 2024; Chen et al., 2024). Spatially, ROIs such as the M1, SMA, or PFC are defined from channel arrays, and activation maps are generated using t-statistics or z-scores (Kim et al., 2023; Kim H. et al., 2022). Functional connectivity (FC) is computed via Pearson correlations (Li H. et al., 2023; Kong et al., 2023) or wavelet phase coherence (Liu L. et al., 2022) between pairs of channels or ROIs, while effective connectivity (EC) can be inferred with Granger causality (Bu et al., 2023; Zou et al., 2024) or transfer entropy (Jian et al., 2021b) to assess directional information flow. Finally, lateralization indices (LI) quantify hemispheric asymmetry (Chen Y. F. et al., 2023; Yuan et al., 2022), and graph-theory metrics (e.g., global efficiency, clustering coefficient) summarize large-scale network properties across the fNIRS-derived connectome (Huo et al., 2023; Yuan et al., 2022; Xu G. et al., 2022).

Clinical metrics in the literature systematically quantify motor, cognitive, and daily-life outcomes, anchored by the Fugl-Meyer Assessment (FMA) for limb motor recovery (Chatterjee et al., 2019; Jian et al., 2021a; Tamashiro et al., 2019), the NIH Stroke Scale (NIHSS) for neurologic deficit (Huo et al., 2023; Yang et al., 2024), and the Barthel Index for activities of daily living (Liu L. et al., 2022). Complementary scales include the Montreal Cognitive Assessment (MoCA) for cognition (Liu Y. et al., 2024), the Western Aphasia Battery (WAB) for language (Xie et al., 2022a; Gilmore et al., 2021), and specialized batteries such as the 10-Meter Walk Test for gait (Chatterjee et al., 2019; Lee A. et al., 2020) and the penetration-aspiration scale (PAS) for swallowing safety (Ma X. et al., 2024; Wen et al., 2023). These validated instruments provide objective benchmarks that enable clinicians and researchers to track progress, stratify patients, and correlate behavioral gains with underlying neuroplasticity measured by fNIRS.

## 4 Future research directions

Current fNIRS paradigms often rely on overly simplified tasks that lack ecological validity, limiting real-world relevance. Most fNIRS paradigms still employ laboratory-centric tasks—such as single-joint finger taps or word lists—that poorly reflect the multisensory, goal-directed demands of daily life after stroke. This ecological shortfall is compounded by low sensitivity: fixed block lengths and uniform difficulty can mask subtle, clinically relevant changes in patients with heterogeneous lesion patterns. Robustness is weakened by uncontrolled motion artifacts and by the absence of harmonized optode montages, leading to high inter-site variability. Furthermore, language paradigms are almost exclusively English-centric, limiting cross-linguistic validity. Finally, few protocols incorporate real-time physiological noise suppression, so cardiac or respiratory drift can masquerade as neural signal. Until these validity, sensitivity, and robustness issues are explicitly mitigated, clinical uptake will remain tentative.

Future fNIRS research on stroke rehabilitation task paradigms should focus on breaking through the following directions: first, to address the highly heterogeneous nature of functional impairments in stroke, it is necessary to develop a more intelligent and adaptive task paradigm design and realize dynamic and personalized task regulation based on individual neurological functional status using artificial intelligence and big data technology. Second, due to its limited spatial resolution (~2–3 cm) and shallow penetration depth, fNIRS cannot resolve activity in small or deep cortical regions, its millisecond-level sluggishness makes it less suitable than electroencephalogram (EEG) for tracking rapid neural dynamics. A multidimensional and cross-modal neurological function assessment system should be constructed, integrating fNIRS with EEG, electromyography, and other multi-source neuroimaging data to comprehensively analyze the brain network remodeling mechanism. Third, introducing VR and AR technologies and designing interactive tasks closer to daily life situations can improve the ecological validity of the task paradigm and enhance patient participation and training transfer effects. Fourth, promote the construction of a standardized task paradigm library, formulate unified task parameters, presentation standards, and data processing norms, and promote the comparability and result promotion of multicenter studies. Fifth, explore the innovative application of closed-loop neurofeedback and brain-computer interface technologies in rehabilitation tasks to achieve real-time neurofunctional monitoring and personalized feedback regulation. Finally, moving fNIRS from bench to bedside is slowed by its high cost, bulky cap/optode assemblies, and the need to train clinicians not only in neuroimaging theory but also in the nuanced interpretation of hemodynamic traces within noisy clinical environments. Beyond the clinic, its utility is further circumscribed by motion artifacts in ambulatory patients, limited depth penetration in adults with thick scalp/skull, and the absence of standardized normative databases for rapid bedside decision-making. Thus, multidisciplinary collaboration is needed to promote the translation of fNIRS technology from scientific research to clinical practice, which ultimately serves to improve functional recovery and quality of life in patients with stroke.

## 5 Conclusion

This paper systematically reviewed the latest research progress of fNIRS in stroke rehabilitation task paradigms, focusing on the design principles of motor, cognitive, language, swallowing, and dual-task paradigms and their neurological mechanism analysis value. This study provides a valuable reference for deepening the understanding of the fNIRS task paradigm in stroke rehabilitation neuroscience research and the subsequent design of more scientific, standardized, and clinically usable fNIRS assessment protocols. In the future, it is necessary to integrate multimodal technologies (e.g., real-time neurofeedback, VR) to construct a dynamic assessment framework and design a personalized paradigm based on injury pattern and recovery stage to optimize closed-loop of assessment-intervention-validation and promote an in-depth translation of the fNIRS from mechanism research to the clinical practice of precision rehabilitation.

## Data Availability

The datasets analyzed during the present study are publicly available in the published articles included in this systematic review. All references and data sources are listed in the manuscript and can be accessed through public databases such as PubMed and Embase.
